# Closed Loop Interactions between Spiking Neural Network and Robotic Simulators Based on MUSIC and ROS

**DOI:** 10.3389/fninf.2016.00031

**Published:** 2016-08-03

**Authors:** Philipp Weidel, Mikael Djurfeldt, Renato C. Duarte, Abigail Morrison

**Affiliations:** ^1^Institute for Advanced Simulation, Theoretical Neuroscience and Institute of Neuroscience and Medicine, Computational and Systems Neuroscience and Jülich Aachen Research Alliance BRAIN Institute I, Jülich Research Center and Jülich Aachen Research AllianceJülich, Germany; ^2^PDC Center for High Performance Computing, KTH Royal Institute of TechnologyStockholm, Sweden; ^3^Faculty of Biology, Albert-Ludwig University of FreiburgFreiburg im Breisgau, Germany; ^4^Bernstein Center Freiburg, Albert-Ludwig University of FreiburgFreiburg im Breisgau, Germany; ^5^Simulation Laboratory Neuroscience – Bernstein Facility for Simulation and Database Technology, Institute for Advanced Simulation, Jülich Aachen Research Alliance, Jülich Research CenterJülich, Germany; ^6^Faculty of Psychology, Institute of Cognitive Neuroscience, Ruhr-University BochumBochum, Germany

**Keywords:** neural network simulations, robotic simulations, neurorobotics, closed-loop, real-time

## Abstract

In order to properly assess the function and computational properties of simulated neural systems, it is necessary to account for the nature of the stimuli that drive the system. However, providing stimuli that are rich and yet both reproducible and amenable to experimental manipulations is technically challenging, and even more so if a closed-loop scenario is required. In this work, we present a novel approach to solve this problem, connecting robotics and neural network simulators. We implement a middleware solution that bridges the Robotic Operating System (ROS) to the Multi-Simulator Coordinator (MUSIC). This enables any robotic and neural simulators that implement the corresponding interfaces to be efficiently coupled, allowing real-time performance for a wide range of configurations. This work extends the toolset available for researchers in both neurorobotics and computational neuroscience, and creates the opportunity to perform closed-loop experiments of arbitrary complexity to address questions in multiple areas, including embodiment, agency, and reinforcement learning.

## 1. Introduction

Studying a functional, biologically plausible neural network that performs a particular task is highly relevant for progress in both neuroscience and robotics. The major focus on this topic in the field of robotics consists of using of neural networks of varying degrees of complexity for controlling robots. So far, the majority of research has focused on non-spiking, artificial neural networks for this task (Antonelo et al., 2007; Dasgupta et al., 2013; Quiñonez et al., 2015), but there is considerable interest in investigating the capacities of spiking neural networks.

In computational neuroscience, the study of simulated neural networks is paramount to gain a better understanding of the processes underlying learning/adaptation to complex environments and global behavior (e.g., Chorley and Seth, 2008). However, neural network simulators do not typically include functionality for representing environments and sensory input. As a consequence, most tasks used to test the function of a simulated neural network are hardcoded to represent a highly specific task. This has the disadvantage that virtual experiments are complex and time consuming to develop and adapt. More importantly, tasks defined in this way are rather artificial (Potjans et al., 2011; Jitsev et al., 2012; Frémaux et al., 2013; Legenstein and Maass, 2014; Friedrich and Lengyel, 2016). Whereas there is certainly value in investigating very simplified tasks and sensory representations, it is also vital to be able to check that proposed neural architectures are capable of handling richer, noisier, and more complex scenarios.

However, design of environments, representation of sensory states and conversion of motor commands into movements are primary features of robotic simulators. Therefore, it would be of great value to both research fields if the powerful tools developed for robotic simulation and spiking neural network simulation could be made to work together. This would allow researchers from both fields to perform simulated closed loop experiments with flexible experiment design, rich sensory input, complex neuronal processing, and motor output. The major challenge lies in the fact that robotic simulators communicate via continuous data streams, while neural network simulators communicate with spike events (so as their biological counterparts). Thus, a principled approach for bi-directional conversion is required.

So far there have been few attempts to address this. Moren et al. (2015) describe a technical setup for a specific use case, in which a robot is connected to a neural network model of early saccade movement simulated with the Neural Simulation Tool (NEST: Gewaltig and Diesmann, 2007). As the robot is not in the same location as the cluster where the neural simulation is running, the connection is realized via the internet using an SSH tunnel and the Multi-Simulation Coordinator (MUSIC: Djurfeldt et al., 2010), a middleware facilitating communication between different neural simulators in one simulation. Using a minimal model and optimal number of cores, the NEST simulation ran a factor of two slower than real time and achieved an output response of 110–140 ms. Although this study provides a proof of concept for interaction between robotics and neural simulators, it does not represent a general solution, in part due to the limited implementation detail provided.

A more general solution was presented by Hinkel et al. (2015), who describe an interface between the Robotic Operating System (ROS: Quigley et al., 2009) and NEST. ROS is the most popular middleware for the robotic community, with interfaces to many robotic simulators and also robotic hardware. It allows the user to create their own simulated robots in great detail using the Unified Robot Description Format. Similarly, NEST is one of the most commonly used neural simulators for spiking point neuron models. However, the proposed interface is on the basis of Python: the neural network must be simulated in brief periods in an external loop, reading out and communicating the spikes to the robotic simulator at each iteration. Although no performance data are provided, the overheads inherent in repeatedly stopping and starting NEST imply severe performance limitations for this approach.

In this paper we present an alternative approach based on ROS and MUSIC that is both general and efficient, enabling real-time performance for up to hundreds of thousands of neurons on our hardware. Our approach provides roboticists the opportunity to extend their work on neural control into the realm of spiking neural networks using any neural simulator implementing a MUSIC interface, including NEST or NEURON (Hines and Carnevale, 2001). Conversely, our toolchain frees the researcher from the constrictions of the hardcoding approach described above, by enabling neural simulators to be connected to any of the myriad robotic simulators implementing a ROS interface (including Gazebo^1^, Morse^2^, or Webots^3^). To demonstrate the capabilities of the toolchain, we implement a Braitenberg Vehicle (Braitenberg, 1986) in which the agent is simulated in Gazebo and the neural controller in NEST. A pre-print version of this manuscript is available on arXiv (Weidel et al., 2016).

### 1.1. Description of the toolchain

The main purpose of the present toolchain is to capture a broad set of use cases by connecting robotic with neural simulators in a generic way, obtained by interfacing the two middlewares (ROS and MUSIC). The interface is licensed under GPLv3 and available on Github as a plug-in for MUSIC^4^.

In this work, we propose a set of possible solutions to overcome the problem of converting between continuous and spiking signals. In particular, we investigate the performance of three different kinds of encoders and a linear readout decoder (see 2.2). As the correct encoding mechanism is debatable and dependent on the scientific question being addressed, in addition to our own mechanisms, we provide extensibility such that researchers can implement their own custom encoders, decoders and adapters in Python or C++.

**Figure 1:**
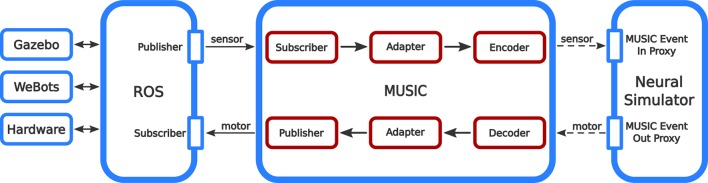
**Information flow in the ROS-MUSIC toolchain**. The boxes with red outlines depict our extensions to MUSIC that facilitate the bidirectional conversion of continuous and spiking signals (solid and dashed arrows, respectively).

To achieve these ends, our interface to ROS extends MUSIC by three different kind of binaries: adapters, encoders, and decoders (see Figure 1). Encoders and decoders are used to translate between spiking data and continuous data while adapters can be used for pre-processing data and connect to ROS. All binaries run in their own process and solve only one specific purpose. This way, the interface is highly modular and the implementation of custom adapters, encoders, or decoders is as simple as possible.

## 2. Materials and methods

### 2.1. The MUSIC configuration file

The adapters, encoders, decoders and simulators introduced in the toolchain are specified, connected and parameterized in the MUSIC configuration file. Detailed information about the MUSIC configuration file can be found in the MUSIC manual^5^ and examples of complete configuration files for this toolchain can be found in the Github repository.

### 2.2. Encoding, decoding, and adaptation

The choice of encoder and decoder to convert continuous to spiking signals is non-trivial, and potentially dependent on the scientific question or task. Here, we describe three possible encoding mechanisms and one decoding mechanism. We also describe a simple adapter to map the dimensionality of the input signal to the dimensionality of the receiving network.

#### 2.2.1. Regular rate coding

The simplest way to encode continuous data into spiking data is regular rate coding. i.e., mapping the input signal to a time-dependent firing rate variable. Each input dimension is encoded by a single neuron by transforming the input signal, which is a continuous signal in the range [−1, 1], into a time-dependent variable representing the spike density of the encoding neuron. This is achieved by calculating the interspike interval of each encoding neuron at time *t* by:

(1) $$ISI_{n}(t)=\frac{1}{v_{\text{min}}+(v_{\text{max}}-v_{\text{min}})\frac{1+I_{n}\left(t\right)}{2}}$$

where *I*_*n*_(*t*) is the input which is mapped to neuron *n*. The firing rate is scaled between [*v*_min_, *v*_max_] which are free parameters of the regular rate encoder. This encoding mechanism leads to very regular spike patterns but has the clear advantage of being computationally very efficient. Note that this approach does not take into consideration biological neuron properties such as refractoriness, but these could be included without much impact on the computational efficacy.

#### 2.2.2. Poisson rate coding

In this encoding scheme, we follow a similar implementation to that described in the previous section, but introduce a stochastic component in the generated spike trains, by transforming the input signal into a time-dependent variable representing the intensity of a stochastic point process with Poissonian statistics. This can be achieved by using the inverse of the expression ((1)) for interspike interval in the previous section as the rate parameter for an exponential ISI distribution:

(2) $$ISI_{n}(t)=-\frac{1}{v_{\text{min}}+(v_{\text{max}}-v_{\text{min}})\frac{1+I_{n}\left(t\right)}{2}}\ln(r)$$

where *r* is a random number drawn from a uniform distribution in [0, 1) and *I*_*n*_(*t*) is the input mapped to neuron *n*. The firing rate is scaled between [*v*_min_, *v*_max_] which are free parameters of the Poisson rate-encoder. This encoding mechanism produces spike trains with Poissonian statistics, similar to those typically assumed to occur in central neurons in the mammalian brain (see for example, Calvin and Stevens, 1967; Dayan and Abbott, 2001; Averbeck, 2009 for a counter-argument), and therefore are thought to be a more realistic approximation than regular spike trains. As Section 3 shows, the computational complexity of this approach is comparable to that of regular rate encoding.

#### 2.2.3. Neural engineering framework

The Neural Engineering Framework (NEF: Eliasmith and Anderson, 2004) is a generic formalism to represent stimuli with neural ensembles that allows a wide variety of functions to be realized by deriving optimal projections between neural populations. In the simplest formulation, NEF encodes a vector of continuous data *I* as an input current to an integrate-and-fire neuron whose activity is defined by

(3) $$a_{n}(I)=G_{n}[\alpha_{n}\cdot I+I_{n}^{\text{bias}}]$$

where *G* represents a generic non-linear functional which is determined by the neuron dynamics, α_*n*_ is the neuron's response preference, or tuning curve, and $I_{n}^{\text{bias}}$ a constant bias current setting the base activation of neuron *n*. It is worth noting that this encoding does not depend on the specific form of *G* and any neuron model can be used for this operation. The original signal *I* can be adequately reconstructed (depending on the properties of G and alpha) by linearly combining the activity *a* of the encoded representations

(4) $$\hat{I}=\sum_{n}a_{n}(I)\phi_{n}$$

where the decoding weights ϕ are obtained by linear regression in order to minimize the reconstruction error.

#### 2.2.4. Linear decoder

Having described possible options explored in this work to convert continuous signals into discrete spike event trains, we now explore the reverse operation. To transform the spiking activity of a given neural ensemble to a continuous signal, which can be used, for example, to provide motor commands to the simulated robot, we first perform a low-pass filter of each spike train by convolving with a causal (i.e., only defined for *t* ≥ 0) exponential kernel *k*(*t*) = exp (−*t*∕τ_dec_) with time constant τ_dec_

(5) $$a_{n}(t)=\sum_{i}k(t-t_{i,n})$$

where *t*_*i, n*_ represents the *i*-th spike time of neuron *n*. The resulting activities at time *t* can be linearly combined to obtain a continuous output signal

(6) $$z_{k}=\sum_{n}a_{n}(t)\phi_{nk}$$

where weights ϕ_*nk*_ define the contribution of ensemble neuron *n* to readout unit *k*.

#### 2.2.5. Signal adapter

For the experiments carried out below we implement a simple adapter that maps the *m* dimensions of the input signal to the *n* dimensions of the receiving neurons. Note that this is only necessary for regular and Poisson rate encoding; the NEF encoder already incorporates this functionality by construction. For the performance measurements, each receiving neuron receives all dimensions of the input signal. For the Braitenberg vehicle, the input signal is split up into two hemispheres, each mapped to one of the two controlling neurons. For more complex experiments, a more sophisticated adapter can be implemented.

### 2.3. Performance measurements

In order to be as close as possible to a real use-case, we simulate the whole toolchain while measuring the performance capabilities of the different parts. The agent used for the performance measurements is a four wheeled, mobile robot simulated in Gazebo with an attached virtual laser scanner. The laser scanner has 100 beams with an update rate of 20 Hz and a maximal range of 5 m. We use the SkidSteerDrivePlugin provided by Gazebo, which allows us to steer the robot with a ROS::Twist message, updated with a rate of 20 Hz. During the measurements involving the NEF encoder, we keep the parameters of the integrate-and-fire (IAF) neurons unchanged and simulate these neurons with a resolution of 1 ms using exact integration (Rotter and Diesmann, 1999; Morrison et al., 2007; Hanuschkin et al., 2010). For decoding, we always use a linear readout where the spiking activity is filtered with an exponential kernel with a resolution of 1 ms. The MUSIC adapters run with an update rate equal to the update rate of the sensory input and motor command output. The measurements were executed on one node of a transtec CALLEO 551 cluster and were averaged over five trials with a simulation-time of ten seconds. Measurements involving neural simulators were carried out with NEST 2.8 (Eppler et al., 2015) and NEURON v6.2.

#### 2.3.1. Real-time factor

The sensory input, the motor output and the processing of the sensory data are all updated in parallel but asynchronously. For robotic applications it is crucial that the execution time for processing input data in each time step is less than or equal to the wall-clock time of each time step, meaning the process is running in real-time.

The real-time factor (*RTF*) is calculated by dividing the simulated time, *t*_sim_, by the wallclock time required to run the simulation, *t*_run_.

(7) $$RTF=\frac{{\text{t}}_{\text{sim}}}{t_{\text{run}}}$$

The total time the toolchain needs to perform a simulation can be divided into different components

(8) $$t_{\text{total}}=t_{\text{build}}+t_{\text{run}}+\epsilon$$

where *t*_build_ is the time the toolchain needs for initialization (allocate memory etc.), *t*_run_ is the actual time the toolchain needs to perform the simulation and ϵ is the time which is not covered by *t*_build_ and *t*_run_ (i.e., garbage collection, freeing memory, etc.). In our measurements we clearly separate the buildup phase from the run-time phase by synchronizing the processes after the initialization of the software using an MPI barrier. This allows us to measure *t*_run_ consistently over all processes.

We use this measurement to investigate different aspects of the toolchain's performance, as described below.

##### 2.3.1.1. Dimensionality of the input

The sensory input to a robot can be very complex and high dimensional. The number of neurons needed to encode a multidimensional input strongly depends on the encoding mechanism. In NEF, about 100 IAF neurons per dimension are used when encoding a stimulus in order to be able to decode that stimulus with a root-mean-square error about 1% (Eliasmith and Anderson, 2004). Using rate coding, the number of neurons can be freely chosen. In the example of the Braitenberg Vehicle (Braitenberg, 1986), we use only two neurons for encoding the complete sensory input (see Section 3.2).

To examine the scalability of the sensory input in our toolchain, we measure how many encoding neurons for different encoders we can simulate until the real-time performance breaks down. We use a binary search to find these limits in an efficient way. When measuring the performance with NEST and NEURON, each neuron instantiated by the encoder is matched by a corresponding neuron in the neuronal simulator, which receives the input spike train and repeats it to the decoder as an identical spike train, thus implementing a minimal processing network. In NEST this is realized by the parrot_neuron, a neuron model that emits a spike for each spike received. In NEURON the repeater neurons are of type IntFire1 equipped with very high input synaptic weights and zero refractory time, thus enforcing the neuron to spike after each input spike. For the rate and Poisson encoder, the encoder neurons have a low firing rate between 1 and 2 Hz. Except for NEST (7 processes) and NEURON (40 processes), each process in the toolchain runs on one dedicated process.

##### 2.3.1.2. Bandwidth

Apart from the computational limitations of simulating neurons, communication is another potential bottleneck in this toolchain. Parts of the toolchain are communicating action potentials (events) between different processes (i.e., from encoder to NEST). In order to investigate the influence of the communication on the real-time performance, we use a rate based encoder and measure the real-time factor as a function of the firing rate of the encoding neurons. We choose the amount of neurons close to the border of real-time capability, so that we can see an immediate impact of the firing-rate on the performance of the toolchain.

##### 2.3.1.3. Latency

The latency between sensory input and motor output can be crucial for the robotic application. A fast reaction time is needed for many applications (for example, catching a ball). In order to measure the latency (or reaction time), we artificially disturb the sensory signal of the robotic simulator, switching discontinuously from the minimum to the maximum sensor range. A very simple encoder responds to this change by beginning to produce spikes, which the neural simulator receives over its MUSIC Event In Proxy and repeats its the MUSIC Event Out Proxy (see Figure 1). A decoder responds to the arrival of this spike train by producing a motor command, which is conveyed over ROS to the robotic simulator. The latency of the tool chain is therefore the wall-clock time between the change in the sensory signal and the reception of the motor signal.

##### 2.3.1.4. Overhead

For measuring the input scalability as described above, we use a MUSIC time step equal to the ROS update rate. This minimizes the communication overhead as only new data is communicated. As latency depends on the MUSIC time step, we additionally investigate the communication overhead of the toolchain. To do this, we determine the real-time factor of the toolchain, using a simple rate encoding mechanism, whilst systematically varying the MUSIC time step and the number of simulated neurons.

## 3. Results

### 3.1. Performance

**Figure 2:**
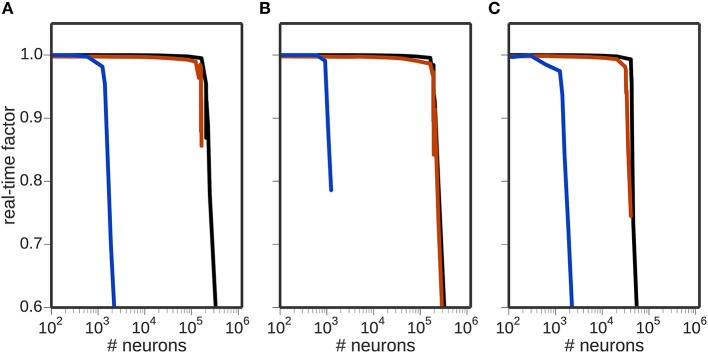
**Scalability of the toolchain**. Real-time factor as a function of the number of neurons used for encoding using different encoders: **(A)** rate encoding mechanism; **(B)** Poissonian encoding mechanism; **(C)** NEF encoding mechanism. In each panel, the black curve shows the real-time performance in the absence of a neuronal simulator, the red curve shows the performance using NEST and the blue curve the performance using NEURON.

Figure 2 shows the effect of the computational load of the different encoding mechanisms on the real-time factor, with and without neural simulators involved in the toolchain. For the rate or Poissonian encoding mechanisms (Figures 2A,B), simulations of up to 150, 000 encoding neurons are possible in real-time when using NEST or when the toolchain does not include a neural simulator. When using NEURON, the real-time factor breaks down at about 1000 neurons. For the NEF encoding mechanism (Figure 2C), simulations of up to 20, 000 encoding neurons are possible in real-time when using NEST or no neural simulator in the toolchain. In this case too, the performance when using NEURON breaks down at about 1000 neurons.

These results demonstrate that the NEF encoder is computationally more expensive than either the rate or Poissonian encoders, and that NEURON is computationally expensive in this context, limiting the real-time performance to 1000 neurons regardless of which encoding mechanism is used.

The breakdown of the real-time performance of the regular rate and Poissonian encoders happens at about the same amount of neurons, which leads to the question what the actual bottleneck of the toolchain is in these cases. Computational complexity is only one limiting factor in this toolchain; another candidate is the communication.

**Figure 3:**
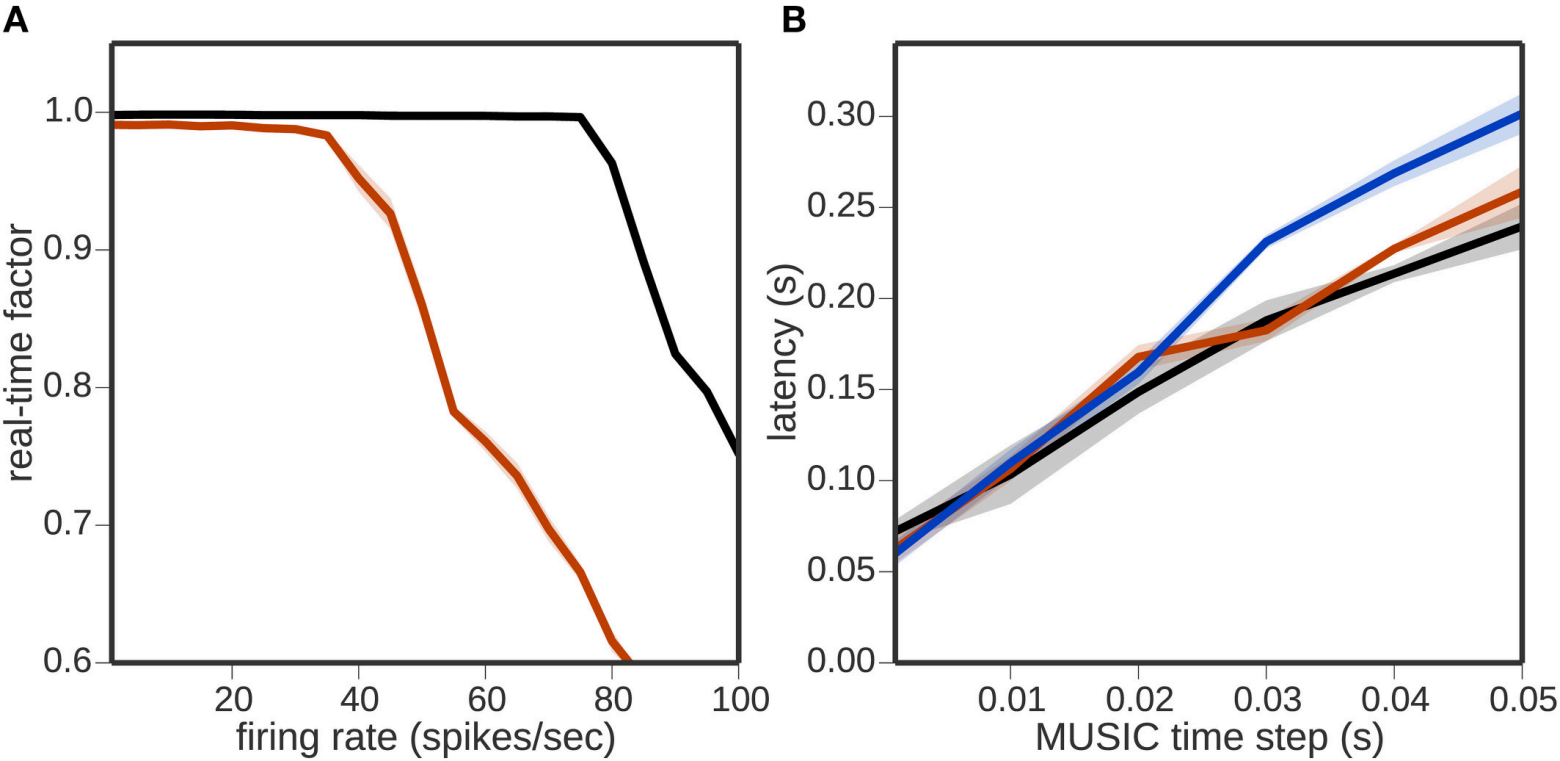
**Bandwidth limitations of the toolchain. (A)** Real-time factor as a function of neuronal firing rate per neuron for a regular rate encoder of 50, 000 neurons without a neural simulator (black curve) and with NEST (red curve). **(B)** Latency as a function of MUSIC time step without a neural simulator (black curve), with NEST (red curve) and with NEURON (blue curve).

But as Figure 3A shows, the real-time factor breaks down at about 40 Hz using 50, 000 encoding neurons. In other words, the toolchain is able to communicate about 2, 000, 000 spikes per second, which is more than was required for the simulations measured in Figure 2. Thus, we can conclude firstly that communication was not the bottleneck for the previous experiment, and secondly that not just the number of the encoding neurons but also their firing rate can be a limiting factor for the real-time performance.

Figure 3B shows the latency of the toolchain, i.e., the difference in time between a change in sensory input and receiving a motor command evoked by that change (see 2.3.1). The latency increases linearly with the MUSIC time step; a MUSIC time step of 1 ms, results in a latency of about 70 ms, growing to around 350 ms for a time step of 50 ms when using a neural simulator. In general, the presence of a neural simulator in the toolchain increases the latency slightly, independently of which simulator is chosen. Note that, for this experiment, an extremely simple encoder and decoder were chosen that have no intrinsic time constants (see 2.3.1.3). In practice, choosing more sophisticated encoders/decoders with long time constants can increase the time it takes for a change on one side of the toolchain to become effective on the other.

**Figure 4:**
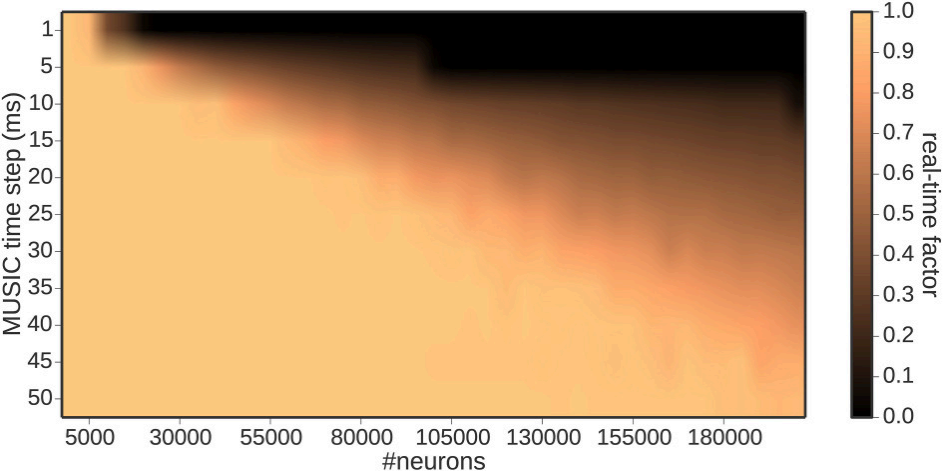
**Real-time factor as a function of the MUSIC time step and number of encoding neurons**.

Figure 4 depicts the dependency of real-time capability of the toolchain on the MUSIC time step. The border of real-time simulation capability increases linearly with the MUSIC time step, from 10, 000 neurons for a time step of 1 ms to 185, 000 neurons for a 50 ms time step. However, as demonstrated in Figure 3B, the latency also increases with the MUSIC time step. From these results, we conclude that the latency and the dimensionality of the input are two conflicting properties, which have to be balanced for the specific use case.

**Figure 5:**
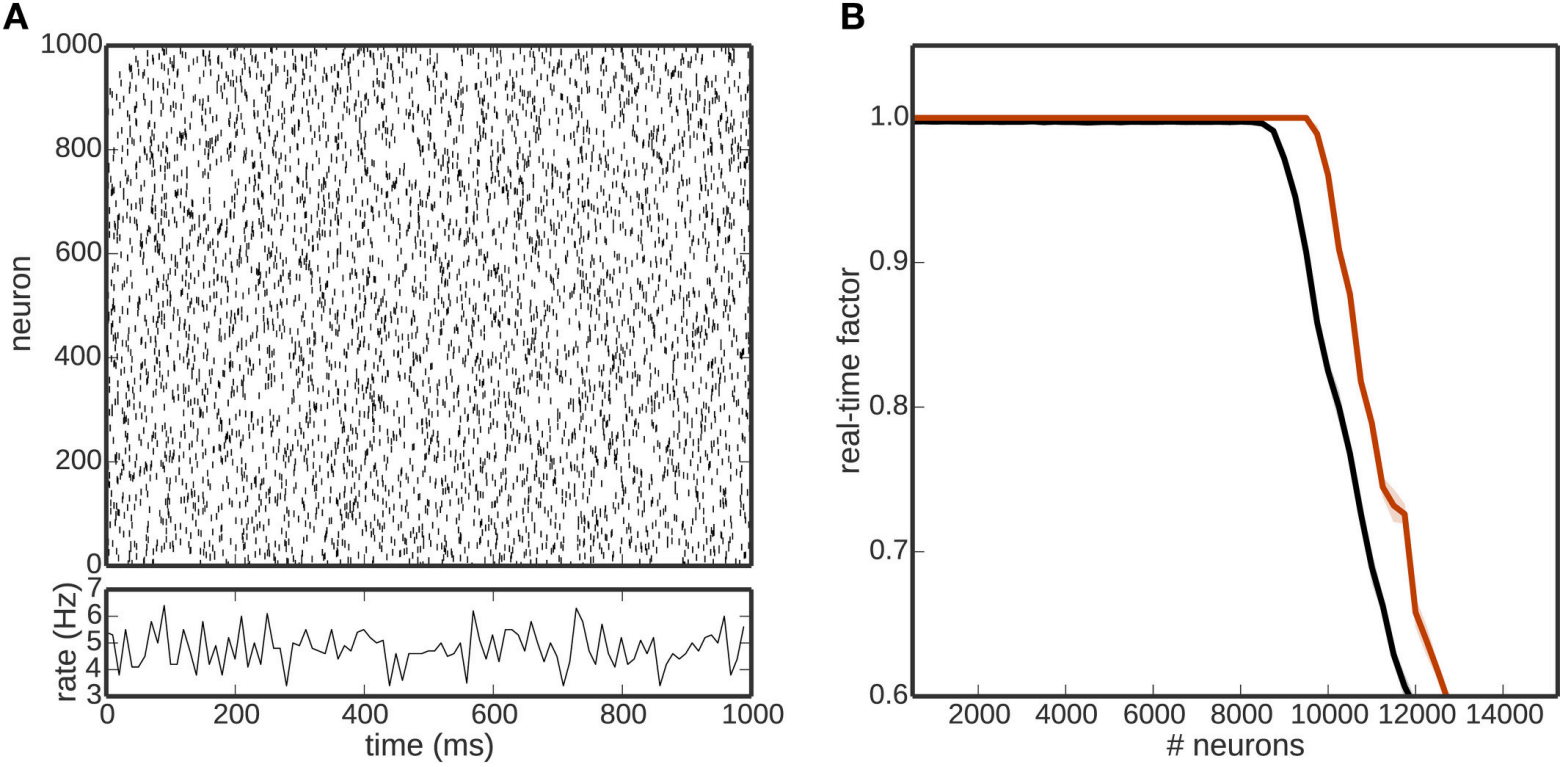
**(A)** Network activity of one realization of the excitatory population of a balanced random network. **(B)** Real time factor as a function of the number of network neurons for a simulation purely in NEST (red) and incorporating the whole toolchain (black). Shaded areas denote the standard deviation over five runs.

In addition to the number of encoding neurons as investigated in Figure 2, a crucial factor for the overall performance of the toolchain is the computational load due to the neuronal network that is receiving and processing the encoded stimuli. In general, the computational load of the neural network simulator is affected by many factors, including (but not limited to) the average firing rate of the network, the choice of neuronal and synapse model, and the simulation time step. In order to get some insight into the limits of the toolchain presented here despite these caveats, we examine the performance in dependence on the size of a neuronal network composed of 80% excitatory and 20% inhibitory leaky integrate-and-fire neurons with delta synapses. This network is integrated with a time step of 1 ms and the number of encoding neurons is kept constant (*N* = 1000; rate encoding) as the network size increases. To ensure comparable network activity at all network sizes, the recurrent synaptic weights are kept very small (*w* = 0.001 mV) and the network is driven by external independent Poissonian spike trains to fire in the asynchronous irregular regime with an average firing rate of around 5 spikes∕s Figure 5A. The results, shown in Figure 5B, demonstrate that the presence of the rest of the toolchain decreases the size of the network that can be simulated in real time by a small but non-negligible amount, i.e., from 10, 000 to 9000 neurons. A smaller choice for the number of encoding neurons would have the effect of shifting the black curve (complete toolchain) closer to the red curve (NEST only), whereas a choice of parameter that increases the computational load of the simulation, e.g., a smaller step size or higher average firing rate) would have the effect of shifting both curves to the left. Clearly, the dominating factor for the overall performance of the toolchain is that of the neural simulator. Consequently, if larger real-time networks are aimed at, either the speed of the neural simulator needs to be improved, or more efficient hardware must be chosen for its execution.

### 3.2. Implementation of a braitenberg vehicle

**Figure 6:**
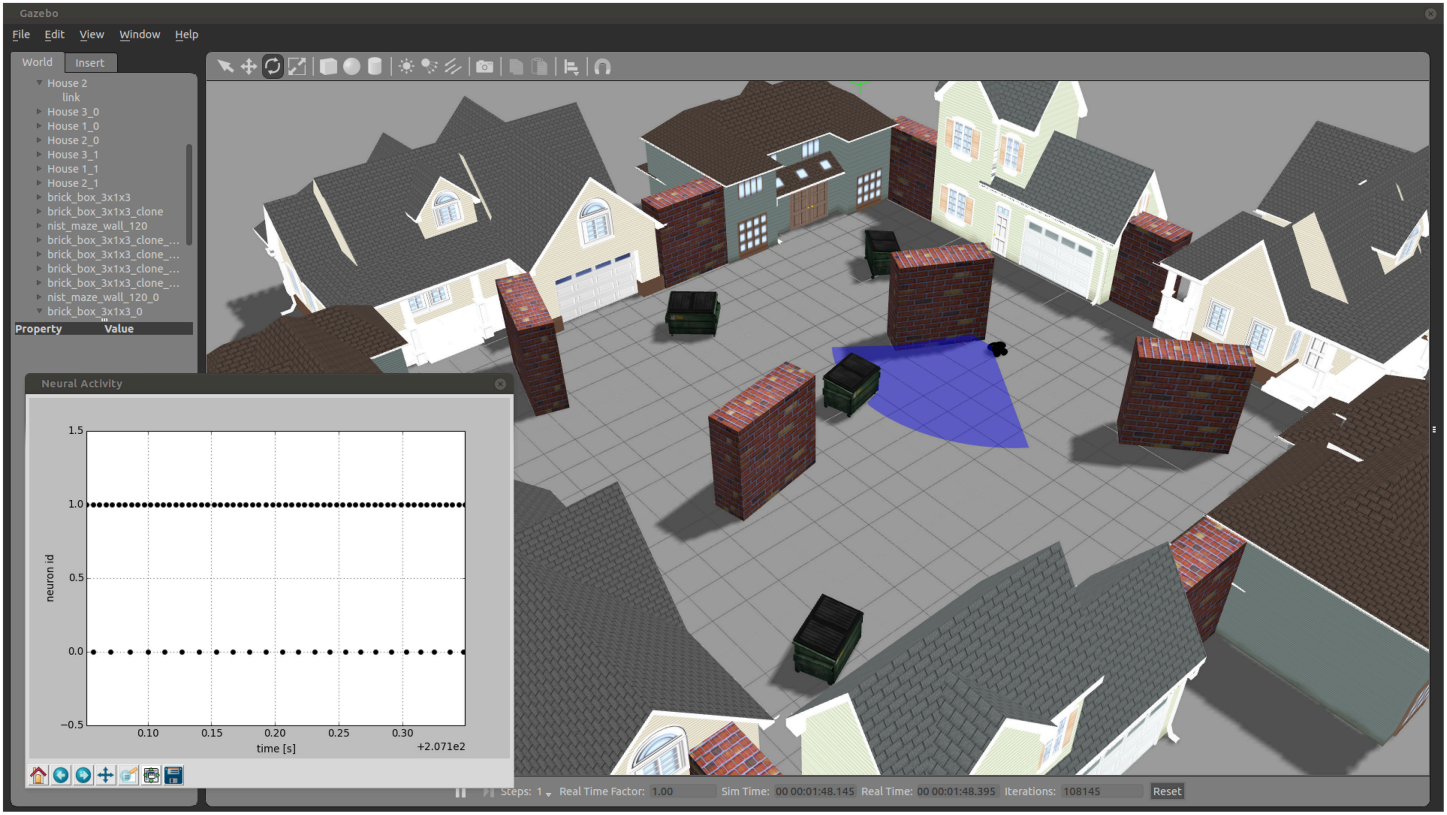
**A Braitenberg Vehicle simulated in Gazebo, controlled by simulated neurons in NEST with the use of the ROS-MUSIC toolchain**. The main figure shows a view of the simulated environment and agent, the sensor range depicted as a blue shaded area. The inset shows the spiking activity of the two controlling neurons.

As an example for the usage of the toolchain, we created a Braitenberg Vehicle III “Explorer” (Braitenberg, 1986) which is simulated in the robotic environment Gazebo (see Figure 6). The Braitenberg Vehicle is implemented as a four-wheeled mobile robot with an attached laser scanner for sensory input. With the use of the ROS-MUSIC toolchain, two neurons, simulated in NEST, control this vehicle to avoid obstacles. A video of the demonstration can be found in the supplementary material and the source code is available on GitHub^6^.

## 4. Discussion

We have described our plug-in for MUSIC, which allows any neuronal network simulator implementing a MUSIC interface to communicate with any robotics simulators implementing a ROS interface. The plug-in converts continuous signals from a robotics simulator into spike train signals for a neuronal network simulator and vice versa. We showed that the toolchain allows real-time performance for a wide range of configurations and provided a simple working example. Additionally, we showed that the proposed toolchain only marginally affects the real-time performance of the neural simulator. In the following, we discuss limits and perspectives for this approach.

### 4.1. Performance

Dependent on the choice of encoder, our toolchain allows the simulation of around 20,000 neurons with a NEF encoder and up to 150,000 neurons with a regular rate or Poisson encoder and achieve real-time performance. Using the NEF encoder, it is recommended to have at least 100 neurons per input dimension in order to encode and decode stimuli with root-mean-square errors of less than 1% (Eliasmith and Anderson, 2004), meaning that our toolchain can encode a 200-dimensional input with NEF in real time on a single process of our hardware.

If a higher dimensional input is required, there are two solutions at hand. First, the NEF encoder could be parallelized and run in more than one process. Second, if a less sophisticated but computationally cheaper way of encoding is acceptable for the scientific question at hand, we also provide regular rate and Poissonian encoding mechanisms. A previous neurorobotic interface implemented the communication between ROS and the neural simulators on the Python level (Hinkel et al., 2015). Although no performance data were published in that study, it is clear that the performance would be strongly limited by this approach.

Another critical characteristic of the toolchain is the latency between sensory input and motor command output of the toolchain, or in other words the reaction time. To minimize the overhead and avoid repeated communication of the same data, it makes sense to set the MUSIC time step equal to the sensory update or motor command rate. However, Figure 3 demonstrates that this leads to a rather high latency. The architecture of communicating processes currently carries an unavoidable source of latency, since data is buffered in MUSIC for every pair of communicating processes. In any chain of communicating processes, the latency due to buffering will thus grow in proportion to the length of the chain. In future work, this can be tackled by combining multiple adapter, encoder and decoder steps in a single process, thus simultaneously minimizing communication and reaching a lower latency, and/or by introducing non-buffered inter-process communication. Combining adapters, encoders and decoders can be realized using a plug-in adapter API which maintains independence between the software components.

### 4.2. Complexity of neuronal simulations

In our measurements of the toolchain incorporating NEST and NEURON, we used only minimal networks solving no particular task. However, neural network models can rapidly become computationally complex, especially when incorporating synaptic plasticity, large network sizes, or multi-compartment neuron models. Such models would be impossible to run in real-time using the current approach. In some cases this issue could be solved by increasing the computational resources, using a cluster or supercomputer (Helias et al., 2012; Kunkel et al., 2014). However, this does not guarantee real-time execution, as neuronal network simulators do not have perfect scaling, due to serial components in their algorithms and communication overhead. Even if the problem can theoretically be addressed in real-time by increasing resources, it might not be feasible to access the quantity required. An alternative prospect is offered by neuromorphic hardware, i.e., hardware purpose built for simulation/emulation of neuronal networks. Two examples that are being developed within the framework of the Human Brain Project are SpiNNaker (Furber et al., 2013) and NM-PM1 (Schemmel et al., 2010), which have the potential to speed up the neuronal simulation massively. In particular SpiNNaker has great potential for neurorobotic applications and an interface between SpiNNaker and MUSIC is already in the prototype phase. This development enhances the value of the toolchain we describe for the neurorobotic community.

Whereas the real-time property is important for robotic control, it is not nearly so important for addressing questions in the field of computational neuroscience. Here, the advantage of the toolchain is that a neuronal network simulation can be provided with rich sensory input from an agent interacting with an environment that is easy for the researcher to configure. In this case, arbitrarily computationally demanding networks can be coupled with robotics simulators simply by slowing down the latter to compensate—gazebo, for example, provides a parameter to conveniently control the time scaling.

### 4.3. Applications

The toolchain we describe gives researchers in computational neuroscience the possibility to test their hypothesis and models under more realistic conditions of noisy sensory input, and researchers in neurorobotics the opportunity to investigate more realistic neurally based controllers. One area of potential interest is the ability to construct interactions between robotic simulators and hybrid neuronal simulations on multiple scales, e.g., a network of point neuron models into which detailed biophysical models are embedded, simulated by NEST and NEURON respectively. This demonstrates a further advantage of the MUSIC-based interaction over pairings of particular simulators or the Python-based interaction presented by Hinkel et al. (2015).

Moreover, our toolchain is particularly well suited for studying closed-loop scenarios, where the neural network receives stimuli from a complex environment and produces an output, which in turn causes the robotic agent to perform actions within that environment. For example, a robotic agent can be placed in a classic experimental set-up like a T-maze and the behavior of the robot adapted by a neurally implemented reinforcement learner (Potjans et al., 2011; Jitsev et al., 2012; Frémaux et al., 2013; Friedrich and Lengyel, 2016). Here, there is a clear advantage over studying such questions just using neural simulators, as the representation of an external environment as a collection of neural recorders and stimulators is complex, and difficult to either generalize or customize. By separating the concerns of environmental, motor, and sensory representation from those of neural processing, our toolchain provides a highly flexible and performant research approach.

## Author contributions

Concepts developed by PW, MD, RD, AM. Design of software by PW, MD, AM. Implementation and experiment design by PW, MD. Experiments carried out by PW. Paper written by PW, MD, RD, AM.

### Conflict of interest statement

The authors declare that the research was conducted in the absence of any commercial or financial relationships that could be construed as a potential conflict of interest.

## Footnotes

^1^http://gazebosim.org/

^2^https://www.openrobots.org/wiki/morse/

^3^https://www.cyberbotics.com

^4^https://github.com/INCF/MUSIC

^5^http://software.incf.org/software/music

^6^https://github.com/INCF/MUSIC
